# The Dental Department of the University of Maryland

**Published:** 1884-11

**Authors:** 


THE DENTAL DEPARTMENT OF THE UNIVERSITY OF MARYLAND.—This institution has commenced its Third Annual Session under the most favorable auspices.

The North, South, East and West, of this country are represented, with students also from France, Belgium, Germany, South America and Australia.

This University Dental Department will comply with the resolutions adopted by the Association of Colleges and National Board of State Dental Examiners and the American Dental Association at their sessions held in August last, and which are to go into operation during the session of 1885–1886. As the sessions of this Dental Department extend from the 1st day of October to the middle of March of each year, the requirements of five months sessions in different years, will entail no change in this respect. All candidates for graduation must after the present session, pass two full sessions of five months each at the University of Maryland Dental Department; the only exceptions to this rule (which is adopted by all the reputable dental schools) being graduations in Medicine with one year's dental pupilage, or one session at a Medical College or University, with two years dental pupilage. No other concession will be made by the dental schools of this country as equivalent for one course of lectures, demonstrations, etc.

While these generally adopted requirements may affect some if the schools which have been rather lax in their requirements for graduation, they will not in any respect conflict with what the faculty if the Dental Department of the

University of Maryland desires, as this school will cheerfully aid in the carrying out of any efforts to maintain and elevate the standard of dental education.

The Editor of the *Independent Practitioner* remarked in the September No. of that Journal, "it" (the Dental Department of the University of Maryland) "has so far as we know, never granted its honors except in course."

